# B cell contribution to immunometabolic dysfunction and impaired immune responses in obesity

**DOI:** 10.1093/cei/uxac079

**Published:** 2022-08-12

**Authors:** Kristine Oleinika, Baiba Slisere, Diego Catalán, Elizabeth C Rosser

**Affiliations:** Department of Internal Diseases, Riga Stradins University, Riga, Latvia; Program in Cellular and Molecular Medicine, Boston Children’s Hospital, Harvard Medical School, Boston, MA, USA; Department of Doctoral Studies, Riga Stradins University, Riga, Latvia; Joint Laboratory, Pauls Stradins Clinical University Hospital, Riga, Latvia; Programa Disciplinario de Inmunología, ICBM, Facultad de Medicina, Universidad de Chile, Santiago, Chile; Centre for Adolescent Rheumatology Versus Arthritis at UCL, UCLH and GOSH and Department of Rheumatology, Division of Medicine, University College London, London, UK

**Keywords:** B cells, obesity, chronic inflammation, metabolic dysfunction, immune response to infection and vaccination

## Abstract

Obesity increases the risk of type 2 diabetes mellitus, cardiovascular disease, fatty liver disease, and cancer. It is also linked with more severe complications from infections, including COVID-19, and poor vaccine responses. Chronic, low-grade inflammation and associated immune perturbations play an important role in determining morbidity in people living with obesity. The contribution of B cells to immune dysregulation and meta-inflammation associated with obesity has been documented by studies over the past decade. With a focus on human studies, here we consolidate the observations demonstrating that there is altered B cell subset composition, differentiation, and function both systemically and in the adipose tissue of individuals living with obesity. Finally, we discuss the potential factors that drive B cell dysfunction in obesity and propose a model by which altered B cell subset composition in obesity underlies dysfunctional B cell responses to novel pathogens.

## Teaser

Obesity—a major risk factor for multiple disorders, including cardiovascular disease and cancer—has tripled globally in less than 50 years and is associated with a chronic inflammatory response. B cells play a central role in driving obesity-associated meta-inflammation, and here we provide a consolidated summary of human B cell dysregulation and the molecular switches potentially driving this B cell dysregulation. A new frontier within the obesity research field, highlighted by the COVID-19 pandemic, is that obesity impacts the ability to respond to and clear infections and we propose a model by which obesity establishes a new “set-point” in the B cell compartment which contributes to this.

## Introduction: obesity—a global healthcare concern

Obesity is defined as body-mass index (BMI) ≥30 kg/m^2^ and has tripled globally in less than 50 years [1]. Alarmingly, the rate of increase in childhood obesity has been greater in many countries than the increase in adult obesity [2]. It is viewed as a metabolic disorder of fat storage with both genetic (e.g. mutations in molecules involved in adipogenesis) and environmental contributions (e.g. increases in portion sizes and availability of food with high glycaemic index) [3, 4]. Obesity is also associated with metabolic dysfunction and chronic systemic low-level inflammation [5–7].

Our understanding of how the immune system is involved in the pathologies linked to obesity has grown substantially over the past decades. From the initial observation of altered cytokines [8–10] and immune cell phenotypes in individuals living with obesity [11, 12], more recent studies have provided mechanistic insight into how obesity-associated changes in metabolites, nutrients, and hormones can drive immune cell functional impairments [13]. Furthermore, we have gained an appreciation of the interplay between inflammation and metabolic dysfunction. This interaction exacerbates metabolic dysregulation and gives rise to changes in the immune compartment that impact the ability of the body to deal with infectious agents or mount appropriate responses to vaccination [14]. Indeed, obesity is a major risk factor for many disorders, including type 2 diabetes mellitus, cardiovascular disease, fatty liver disease, and cancer [1] and obese individuals experience more severe influenza and SARS-CoV-2 infections and respond poorly to vaccination [15–20]. Alterations within the B cell compartment are a major contributing factor to these outcomes.

Although the contribution of B cells to meta-inflammation and obesity-associated impairments in immunity is now recognized, a consolidated summary of the events is yet to be provided. To address this gap, in this review, we introduce the data from animal models showing that B cells have a direct role in controlling meta-inflammation associated with obesity, before summarizing the emerging studies from humans that demonstrate altered B cell subset frequencies and function in the periphery and adipose tissue of individuals living with obesity. We then discuss data from humans and animal models that uncover potential mechanisms for why there is B cell dysfunction in obesity. Finally, using examples from COVID-19, we propose a model by which the establishment of a new B cell set-point in obesity impairs their ability to respond to vaccination and to help clear infections. Together, we aim to underscore that metabolic dysfunction and B cell abnormalities act in concert to drive pathology and dysregulated immune responses during obesity.

## B cell role in driving obesity-associated metabolic disease—observations from animal models

That B cells are centrally implicated in obesity-associated inflammation and contribute to metabolic dysfunction was first observed in murine models of diet-induced obesity (DIO) approximately a decade ago [21–23]. While B cells are found in adipose tissue in the steady state, feeding a high-fat diet (HFD) to mice was shown to increase trafficking and B cell accumulation in visceral adipose tissue [23]. In addition, B cells were shown to drive adipose tissue inflammation, including the activation of pro-inflammatory macrophages, insulin resistance, and glucose intolerance [21, 22]. These two seminal studies reported that compared to DIO wild-type mice, DIO B-cell deficient mice had lower fasting glucose and insulin levels as well as improved glucose tolerance and insulin sensitivity [21, 22].

More specifically, Winer *et al.* demonstrated that the ameliorated glucose metabolism in obese B-cell deficient mice could be worsened by the transfer of splenic B cells from DIO mice, but not those from mice on a normal chow diet (NCD) [21]. These data suggest that B cells acquire a pathogenic phenotype upon exposure to HFD. B cells play important roles in immunity, which can be categorized into (1) antibody production, (2) antigen presentation to modulate T cell responses, and (3) cytokine secretion, which impacts both other leukocytes and tissue cells [24–26]. Here, B cells were shown to require antigen presentation capacity (expression of MHC molecules) as well as the presence of T cells to exert their pathogenic effects on metabolic parameters following exposure to HFD [21]. Also implicating antibody production as a mechanism of B cell promotion of metabolic disease, in this study, HFD in wild-type mice lead to increased IgG2c, a pro-inflammatory isotype associated also with autoimmunity in murine models, in both visceral adipose tissue (VAT) and serum [21, 27]. That the transfer of IgG but not IgM isolated from the serum of DIO wild-type mice (insulin resistant) to DIO B cell deficient animals was able to induce inflammation and impaired glucose metabolism, suggests an isotype-specific pathogenic mechanism [21]. Building on these findings, DeFuria *et al.* suggested an additional role for B cells in controlling obesity-associated inflammation by regulating T cell pro-inflammatory cytokine production [22].

Interestingly, in both studies discussed above B cells were not involved in the regulation of obesity, as the weight gain on HFD was comparable between B cell deficient and wild-type mice [21, 22]. B cells did however play a role in modulating adipose tissue ‘health’ (see Box 1) [21, 22]. Indeed, DeFuria *et al.* observed that in the absence of B cells, adipocytes were 15% and 40% smaller in VAT and subcutaneous adipose tissue (SAT), respectively; this was also associated with reduced leptin production by adipose tissue explants as well as in serum [22]. Winer *et al.* did not see a difference in adipocyte size between B cell deficient and wild-type mice on HFD but instead observed a decrease in VAT:SAT ratio in B cell deficient mice [21]. Overall lipid metabolism in DIO was also unaffected by B cell deficiency, as serum free fatty acids, triglycerides, and respiratory exchange ratio were comparable to wild-type mice [21, 22].

**Box 1. Adipose tissue in health and obesity**

Adipose tissue is central in the development and shaping of obesity-associated diseases. Excess energy is stored in the form of triacylglycerols in white adipose tissue (WAT) [92]. WAT is composed of adipocytes and the stromal vascular fraction, which also contains leukocytes. In the lean state, the adipose tissue immune cell profile is anti-inflammatory; in obesity it is shifted to a pro-inflammatory state, which has systemic immune consequences. Of note, WAT is further subdivided into SAT and VAT. SAT and VAT differ in structure and function. Importantly VAT contains a larger number of immune cells and is associated with greater morbidity and mortality risk than SAT [93, 94]. The immune landscape of adipose tissue is comprehensively reviewed elsewhere [95].

Notably, B cell depletion with an anti-CD20 monoclonal antibody ameliorated metabolic disease in DIO wild-type mice [21]. Glucose intolerance in DIO mice was similarly ameliorated by targeting B cell activating factor (BAFF) [28], a cytokine important for peripheral B cell maintenance [29]. Together these findings not only underscore the pathogenic role of B cells in meta-inflammation but also suggest that metabolic disease can be modulated by targeting B cells.

In addition to their pro-inflammatory role, certain B cell subsets, collectively known as regulatory B cells (Bregs), can act to suppress immune responses and restrain excessive and pathological inflammation [30]. The hallmark of Breg suppression is the production of the anti-inflammatory cytokine IL-10, but other immunoregulatory mechanisms have been shown to mediate their function [30]. Nishimura *et al*. were the first to identify that in murine adipose tissue a population of B cells constitutively produce IL-10 and are the major source of this cytokine in adipose tissue [31]. This contrasts with B cells from the spleen and lymph nodes, which do not express IL-10 under homeostasis [32]. Adipose tissue Bregs were phenotypically CD19^+^CD1d^lo^CD5^-/lo^CD21^lo^CD23^-/lo^CD25^+^CD69^+^IgM^+^IgD^+^, a phenotype reminiscent of activated or tissue-resident B cells in mice [31]. These Bregs suppressed inflammation and insulin resistance as B cell-specific lack of IL-10 enhanced adipose tissue inflammation and insulin resistance in DIO mice [31]. Furthermore, the Breg fraction was reduced in adipose tissue in obese mice [31]. Abnormalities in glucose metabolism in DIO mice could be ameliorated by the adoptive transfer of total adipose B cells either directly into the subcutaneous fat pads or systemically to DIO B cell deficient mice [31]. Contrary to the two earlier studies described above [21, 22], Nishimura *et al*. observed worsened glucose metabolism in the absence of B cells—it was suggested that this could be driven by environmental factors such as microbiota [31], however these differences between studies are yet to be reconciled.

Shen *et al.* also documented the importance of B cell-derived IL-10 in metabolic homeostasis, however, differing from the study by Nishimura *et al*. these cells were phenotypically CD19^+^CD5^+^ B-1a cells (B-1 B cells can be divided into two functionally distinct subsets, namely, B-1a (CD5^+^) and B-1b (CD5^−^) B cells [33]) [28]. Importantly, subsets of both B-2 and B-1 cells have been shown to secrete IL-10 and have regulatory capacity [30]. In this study, the authors showed that B-1a cells were the main source of IL-10 in the VAT and peritoneal cavity (constituting up to 50% of IL-10^+^ B cells). In DIO mice the frequency of VAT, peritoneal cavity, and splenic IL-10^+^ B-1a cells was reduced compared to lean mice [28]. The transfer of *Il10*^−/−^ B-1 cells also failed to effectively restore glucose homeostasis in DIO B cell deficient mice. In this study, unlike the pathogenic role of IgG antibodies in the context of obesity observed by Winer *et al*. [21], IgM was required for B-1 cells to suppress metabolic disease in DIO mice, since secretory IgM (sIgM)-deficient B-1 cells lost their suppressive capacity [28]. B-1b cells have also been shown to attenuate obesity-associated inflammation and dysfunctional glucose metabolism through the production of IgM [34]. Having previously shown that global deficiency of Id3, a transcriptional repressor that regulates B cell proliferation and activation, ameliorated VAT expansion in DIO [35], in this paper, the authors observed that B cell-restricted Id3 deficiency led to a preferential expansion of B-1b B cells in adipose tissue, which was associated with improved glucose clearance compared to DIO wild-type littermates [34]. Notably, B-1b cells from Id3^−/−^ mice were able to ameliorate obesity-associated inflammation, but B-1b cells from sIgM^−/−^ mice were not.

## Alterations in B cell composition and function in human obesity

Studies from animal models demonstrate a central role for B cells in driving metabolic dysfunction and inflammation in obesity—by an induction of pro-inflammatory B cell phenotypes and a suppression in the regulatory capacities of B cells. These observations formed an impetus to translate these findings into humans and characterize B cell abnormalities in the periphery and adipose tissue of patients living with obesity. However, until recently, B cells in human obesity had remained poorly characterized. Addressing the gap, in 2016 Frasca *et al.* were the first to report a reduced frequency of class-switched memory B cells in the peripheral blood of individuals living with obesity, while the frequency of a subset of B cells termed double-negative (DN) B cells, due to their lack of IgD and CD27 expression, was increased [36] (see Box 2). In a subsequent publication, the authors also observed that DN B cells were further enriched in frequency in obese SAT [37]. Of note, DN B cells are expanded in chronic autoimmune conditions including systemic lupus erythematosus, where they are postulated to give rise to extrafollicular plasmablasts that produce autoreactive antibodies [38]. Supporting a dominant role for IgG-driven meta-inflammation in obesity, and similarly to the data from animal models, peripheral B cells isolated from individuals living with obesity produce increased levels of IgG reactive to SAT protein lysates compared to lean subjects [37]. Production of such autoantibodies was further increased by B cells from obese SAT [37]. Attempting to differentiate between autoantibodies due to increased BMI and autoantibodies due to meta-inflammation in overweight and obese individuals, Winer *et al*. probed the reactivity of serum antibodies against an array of 9000 spotted antigens in obese and overweight individuals, who differed between them in terms of insulin sensitivity [21]. They observed that in obese and overweight individuals 122 and 114 IgG reactivities were distinctly associated with insulin resistance and sensitivity, respectively. The authors found that the targets were mostly intracellular and with ubiquitous tissue expression [21]. Some of these autoantibody targets were also identified in cultures of the stromal vascular fraction obtained from obese individuals by Frasca *et al.*, including the kinase BTK and aspartoacylase [21, 39].

**Box 2. B cell development and differentiation pathways**

The stage of B cell development and mode of activation shaped by the immune context impacts the magnitude and quality of the overall B cell response. B cells egress from the bone marrow as transitional B cells, which migrate to the spleen to give rise to naïve mature B cells, which participate in immune responses [96]. In canonical immune responses following antigen encounter, naïve B cell activation progresses through both the germinal centre (GC) and the EF pathways—each with a different output [97]. Plasma cells produce antibodies maintaining baseline protection, while memory B cells differentiate into antibody-secreting cells after repeated pathogen exposure [97]. In EF responses, double-negative (DN) (IgD^-^CD27^-^) B cells are the proposed intermediate between naïve B cells and the EF plasmablasts, and as such they can be viewed as ‘EF memory’ B cells.

The GC reaction leads to delayed high-affinity, long-lived antibody production and the EF response gives rise to early, low-affinity antibodies [97]. While somatically hypermutated, affinity matured immunoglobulins and memory B cells can be generated through both pathways, the EF response is considered largely T-independent (TI) and not to sustain long-term immune memory [97]. Of note, in T-dependent (TD) responses B cells require CD4^+^ T cell help for antibody production, while in TI responses B cells can generate an antibody response without CD4^+^ T cell help [98].

In mice, B cells are divided into two main ontogenically different populations: B-1 and B-2 cells [99]. In contrast to B-2 cells, which are enriched in secondary lymphoid organs [100], B-1 are enriched at mucosal sites, the omentum [101], peritoneal and pleural cavities, but are also found in the spleen [100]. They are polyreactive with restricted specificity, predominantly IgM-expressing (low rates of class-switching to other antibody isotypes) and produce ‘natural’ immunoglobulins [102], which arise without immune exposure or vaccination [100, 103]. Epitopes often recognized by B-1 cells are conserved patterns expressed on invading pathogens and dying cells [104, 105]. In the absence of infection, they are considered protective due to their involvement in the clearance of host antigens. In humans, CD19^+^CD27^+^CD43^+^ B cells have been suggested to represent the functional counterparts to murine B-1 B cells, however, this remains contentious; others have proposed that these CD19^+^CD27^+^CD43^+^ cells represent an early antibody-secreting cell population rather than a B-1 cell functional equivalent [106].

When further considering the evidence regarding Bregs as critical for controlling adipose tissue homeostasis in animal models, a decrease in peripheral blood transitional B cells and their IL-10 production has also been documented in human obesity [40]. Transitional B cells have been extensively found to be enriched in Bregs, both expressing IL-10 and CD1d [41, 42]. In human SAT, a reduction in relative transcript levels of the pan-B cell marker CD19 as well as IL-10 with increasing BMI has also been reported, suggesting a decrease in anti-inflammatory B cells [31]. Supporting an altered balance of pro- and anti-inflammatory B cells in human obesity, it has also been demonstrated that upon culture with CpG (a ligand for Toll-like receptor 9) and an agonistic anti-BCR antibody, isolated B cells from obese individuals had higher production of IL-6 and lower production of IL-10 compared to lean subjects [36]. B cells from obese individuals also produced more TNF-α *ex vivo* [36].

B-1 B cell and IgM isotype-specific protective roles have also been suggested in association with human obesity. Mirroring their observations in animal models, Harmon *et al*. identified CD19^+^CD27^+^CD43^+^ B cells, the proposed human counterpart to murine B-1 cells [43], in human omental adipose tissue of patients undergoing bariatric surgery [34]. Of the 16 patients with obesity that they collected tissue from, four had a substantial enrichment of these B cells in omental adipose compared to SAT and blood [34]. In line with a role for B-1 cells, there are also indications from human studies that IgM antibodies are protective and regulate obesity-associated inflammation. Indeed, circulating MCP-1 levels [44], previously shown to be highly predictive of insulin resistance, were found to inversely correlate with the amount of both VAT and serum phosphatidylcholine-reactive IgM [34]. However, a correlation between phosphatidylcholine-reactive antibodies and insulin resistance in individuals living with obesity was not observed in this study [34].

## Potential mechanisms-molecular switches that could underlie B cell dysfunction in obesity

**Figure 1.**
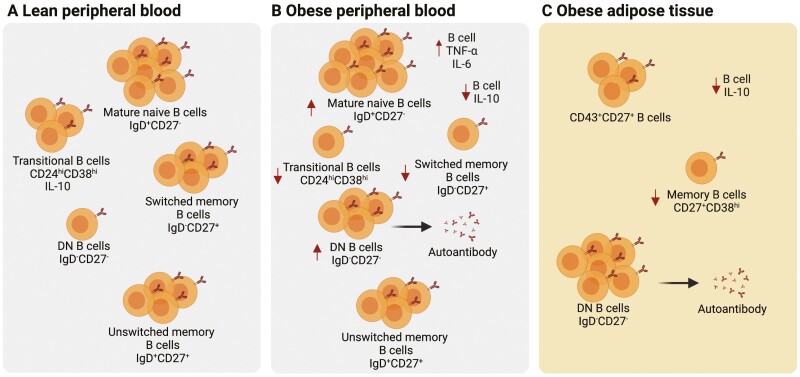
The B cell compartment in lean and obese individuals. **(A)** The B cell compartment in the peripheral blood of lean individuals is composed of naïve B cells (IgD^+^CD27^-^), switched (IgD^-^CD27^+^) and unswitched (IgD^+^CD27^+^) memory B cells and a small population of IgD^-^CD27^-^ double negative (DN) B cells. Among naïve B cells are CD24^hi^CD38^hi^ transitional B cells, which contain IL-10-producing Bregs. **(B)** In patients living with obesity, there are several alterations in the B cell compartment compared to lean individuals. These alterations include an increase in IgD^+^CD27^-^ mature naïve B cells, a reduction in CD24^hi^CD38^hi^ transitional B cells and IL-10 production by these cells. There is also a decrease in class-switched memory B cells and a reciprocal expansion of DN B cells, which have been shown to give rise to autoantibodies. Functionally, it has also been shown that in obesity, B cells can produce more of the pro-inflammatory cytokines TNF-α and IL-6. **(C)** B cell compartment has also been characterized in the adipose tissue of obese individuals. There are reduced frequencies of CD27^+^CD38^-^ memory B cells and IL-10 producing B cells in the adipose tissue of obese compared to lean individuals. DN B cells are further expanded in adipose when compared to circulation of obese individuals. In obese adipose, a substantial fraction of B cells are also CD27^+^CD43^+^ – a phenotype associated with protective IgM responses. However, it is not known if these cells are found in the adipose tissue of lean individuals. Created with BioRender.com.

Considering the strong evidence that obesity is associated with a B lymphocyte compartment that is skewed from anti-inflammatory to pathogenic, it is crucial to consider the mechanistic factors with the potential to alter B cell function in obesity (Fig. 1). Investigation of these potential mechanisms would allow for enhanced possibilities to therapeutically target B cell-driven inflammation in individuals with obesity. Initial evidence suggests a dominant role for adipokines such as leptin, changes to nutrient availability and alterations to the gut microbiota in controlling changes to B cell function in patients living with obesity.

Leptin is a hormone secreted by adipose tissue and its levels are increased in individuals with obesity in a BMI-dependent manner [45]. Leptin bears structural similarity to IL-6 family members and indeed it is now appreciated for its effects on the immune system [46]. B cells express the long signalling isoform of the leptin receptor Ob-Rb [47, 48], and its expression in B cells can be further upregulated by inflammatory signals associated with meta-inflammation in obesity [49, 50]. Increased levels of leptin in individuals living with obesity coupled with the ability of B cells to perceive the adipokine suggest that it may be one of the regulators of the pro-inflammatory/regulatory B cell balance. In support of leptin driving a pro-inflammatory B cell phenotype, Argawal *et al*. demonstrated that *in vitro* leptin had the capacity to induce the secretion of cytokines such as IL-6 and TNF-α by isolated human B cells in a concentration-dependent manner [51]. Leptin also increased B cell expression of CD25 and HLA-DR [51].

Claycombe *et al.* demonstrated that leptin has a role in B lymphopoiesis [52]. Compared to lean wild-type controls, leptin-deficient (ob/ob)-mice had a 60% reduction in nucleated cells in the bone marrow with the B cell compartment the most affected (70% decrease) [52]. Short-term recombinant leptin supplementation led to a significant restoration of B cell numbers [52]. The interpretation of these findings is complicated as leptin-deficient mice become obese—it is possible that the effect of leptin supplementation is indirect. Furthermore, the authors observed that in wild-type mice leptin treatment resulted in a small but significant reduction in B cell numbers which may point to the deleterious effects of excessive leptin on B cells [52]. This suggests that leptin could also drive the depletion of anti-inflammatory transitional B cells.

With regard to the mechanism of how leptin alters B cell phenotypes, metabolic reprogramming of B cells has been proposed. Leptin has been demonstrated to activate the mammalian target of rapamycin complex 1 (mTORC1); this pathway drives protein synthesis, ribosome biogenesis and inhibits autophagy, and is associated with pro-inflammatory immune cell functions [53]. Interestingly, adiponectin levels are reduced in individuals living with obesity [54], and it can stimulate AMP-activated protein kinase (AMPK). AMPK is a key cellular sensor of changes in nutrient availability, activated when energy resources are low [55]. It directs cells towards increased catabolism and decreased anabolism and because of this is the perceived antagonist of mTORC1 [55]. In relation to the potential role of the adiponectin-AMPK pathway in preventing pro-inflammatory B cell activation, the differentiation from metabolically active recent bone barrow emigrant transitional to mature quiescent B cells is accompanied by the induction of AMPK activity [56]. Such B cell maturation is also associated with decreases in mTORC1 activity, glucose uptake, mitochondrial mass, and reactive oxygen species (ROS) production [56]. In support of a model, where leptin and adiponectin have opposing roles in driving pro-inflammatory and anti-inflammatory B cell functions, respectively, B cells from individuals living with obesity have reduced pAMPK compared to lean individuals [36]. Stimulation of isolated B cells from lean individuals with leptin reduced pAMPK levels to those observed in obesity [36]. It was found that pSTAT3 instead was more abundant in B cells from obese individuals compared to B cells isolated from lean; in B cells from lean subjects leptin induced pSTAT3 levels comparable to those observed in obesity [36]. Of, note, it has been previously demonstrated that STAT3 is involved in IL-10 transcription both in mouse and human B cells [57, 58].

In addition to changes in the availability of adipokines, nutrients and metabolites (glucose, free fatty acids) have been demonstrated to modulate B cell function [31, 59] and could affect B cell function in obese individuals. Due to adipose tissue Breg unique constitutive expression of IL-10 compared to other Bregs, Nishimura *et al*. hypothesized that their microenvironment supports these characteristics [31]. They demonstrated that co-culturing murine B cells with SAT increased their IL-10 secretion and survival [31]. Saturated free fatty acids, released from adipocytes during lipolysis, can activate TLR4, a pathway known to stimulate IL-10 production [32]. They indeed showed that palmitate improved survival and IL-10 production among cultured murine adipose tissue B cells; this effect was not observed in splenic B cells [31]. Nishimura *et al*. also showed that IL-10 acts in an autocrine manner to increase B cell viability and IL-10 production in adipose and not in the spleen [31]. Pre-treatment of adipose B cells with IL-10 enhanced their capacity to suppress CD8^+^ T cell CD44 and IFN-γ expression [31]. Jennbacken *et al*. demonstrated that in vitro increasing concentrations of glucose but not insulin or leptin reduced secretion of IgM specific for total LDL, copper oxidized LDL, and malondialdehyde-LDL from TLR4-stimulated mouse B-1 cells [59]. The authors also reported decreased B cell proliferation and differentiation into antibody-secreting cells (ASC), as well as increased apoptosis [59].

A gut-adipose axis has also been suggested [60]. Host-microbiome interactions have been shown to play a role in obesity-associated metabolic inflammation [61]. In addition to microbial dysbiosis, HFD feeding in mice is associated with aberrant mucosal barrier function, subsequent bacterial product leakage, and metabolic endotoxemia in mice—processes in which B cells are also implicated as effectors [62, 63]. Human B cells in turn may be activated by bacteria [36]. Indeed, there is increased expression of TLR4 in circulating unstimulated B cells in obese individuals [36]. While much remains to be addressed about how microbiota impacts B cell function in obesity, we have previously shown that gut microbiota-derived signals expand IL-10-expressing Bregs in mice [57]. Since the gut microbiota of bariatric surgery patients resembles those of lean individuals [64], it would be of interest to determine how bariatric surgery impacts B cell populations in different sites. Notably, it has been demonstrated that IgA-expressing intestinal B cells play a protective role in murine DIO [60]. In the intestinal lumen, IgA is produced mainly in its dimeric form and acts to regulate gut homeostasis [65]. Luck *et al*. observed that in obese mice the frequency and absolute number of IgA^+^B220^-^ ASCs was reduced in intestinal immune sites (colon and the mesenteric lymph nodes) and secretory IgA in the luminal contents was also decreased [60]. IgA was additionally shown to regulate glucose metabolism as in IgA-deficient obese mice glucose tolerance and insulin sensitivity were worsened compared to DIO wild-type animals. The lack of IgA in DIO mice led to changes in the intestinal microbiome, which could be transferred to antibiotics-treated (microbiota-depleted) obese wild-type mice through faecal transplantation. Notably, it has also been demonstrated that oxysterol metabolism is impacted in HFD-fed mice [66], and that this leads to alterations in the differentiation of IgA ASCs within gut-associated lymphoid tissue [67, 68]. More specifically, Trindade *et al*. demonstrated that levels of the oxysterol 25-hydroxysterol were increased within the Peyer’s patches of HFD- versus standard chow-fed mice [67]. This was associated with a reduction in the generation of antigen-specific IgA ASCs. In mice lacking CH25H, a key rate-limiting enzyme in the production of 25-hydroxycholesterol, the impact of an HFD on the differentiation IgA-ASCs was ablated. A key consideration of future research is to generate an integrative model by which the impact of both dietary-induced changes to metabolite availability and the components of gut-microbiota are considered when investigating the impact of obesity on B cell responses.

## Impact of obesity on the response to infection and vaccination—issues highlighted by the COVID-19 pandemic

A new frontier within the obesity research field is an appreciation that as well as meta-inflammation, obesity impacts the ability to respond to and clear infections and mount appropriate vaccine responses. During the COVID-19 pandemic, associations with severe disease were diligently recorded, and obesity emerged as an independent risk factor [15, 18–20], which mirrors findings from the 2009 H1N1 influenza pandemic [69–74]. Furthermore, patients living with obesity have reduced antibody production in response to vaccination, including influenza and hepatitis B [69, 75–78]. The causes for this paradoxical state of chronic inflammation but reduced adaptive responses to novel antigens in obese individuals remain elusive, however, this is likely due, at least in part, to the drastic alterations in the B cell compartment described above.

Murine studies have provided critical support in demonstrating that obesity specifically impacts B cell responses to infection and vaccination [79–83]. DIO mice have increased mortality from infection with the 2009 pandemic H1N1 virus despite comparable viral titres to lean controls [84]. Furthermore, obese mice have both delayed and diminished antibody production in response to infection with influenza A/Puerto Rico/8/34 virus [80]. When challenged with the heterologous pH1N1 virus 5 weeks after, these obese animals had lower levels of cross-reactive nucleoprotein antibodies and fewer mice-generated hemagglutination–inhibiting antibodies compared to lean controls [80]. This was additionally associated with increased lung pathology in DIO [80]. In this study, the mortality of lethal pH1N1 infection was prevented by priming with the A/Puerto Rico/8/34 virus in both obese and lean mice [80]. It has also been demonstrated that increased disease severity in response to *Staphylococcus aureus* infection in obese mice is linked with impaired pathogen-specific antibody class-switching from IgM to IgG [82]. With regard to vaccination in obesity models, Kim *et al*. demonstrated that upon immunization with the commercial 2009 H1N1 vaccine, HFD-fed mice had lower antibody titres and neutralizing activities compared to NCD-fed controls [83]. Importantly, this was associated with reduced protection upon the H1N1 challenge as disease severity and mortality were significantly increased [83]. In fact, mortality following challenge in HFD immunized mice was comparable to that of NCD-fed naïve animals [83]. Together these data from murine studies support the notion that antibody-mediated protection is compromised in obesity with both suboptimal priming and induction of memory. This is also supported by human studies [36, 78]. Reduced antibody titres have been reported in individuals living with obesity 1-year post-vaccination with the trivalent influenza vaccine [78]. Frasca *et al*. found that the antibodies from obese individuals had a lower neutralizing capacity than those from lean subjects approximately 1 month following the influenza vaccine [36]. Of note, the ability to mount peripheral B cell responses in murine models appears to be differentially impacted by obesity based on whether the initial encounter of pathogen-associated molecules occurs through infection or vaccination; this could be because a narrower selection of pathways is engaged through immunization due to more limited epitopes and pathogen-associated molecular patterns available. Interestingly, another study in mice investigated whether adjuvants could be employed to overcome obesity-associated defects in mounting antibody responses to vaccination [81]. The authors found that despite enhanced seroconversion, the breadth (number of epitopes targeted) and magnitude of antibody response remained significantly lower in obese mice compared to lean control animals; obese mice also exhibited delayed viral clearance upon challenge with influenza [81]. The causes for reduced memory to novel pathogens in obesity have not been addressed in-depth, but a reduced frequency of bone marrow CD138^+^ B cells has been demonstrated in obese mice [79].

**Figure 2.**
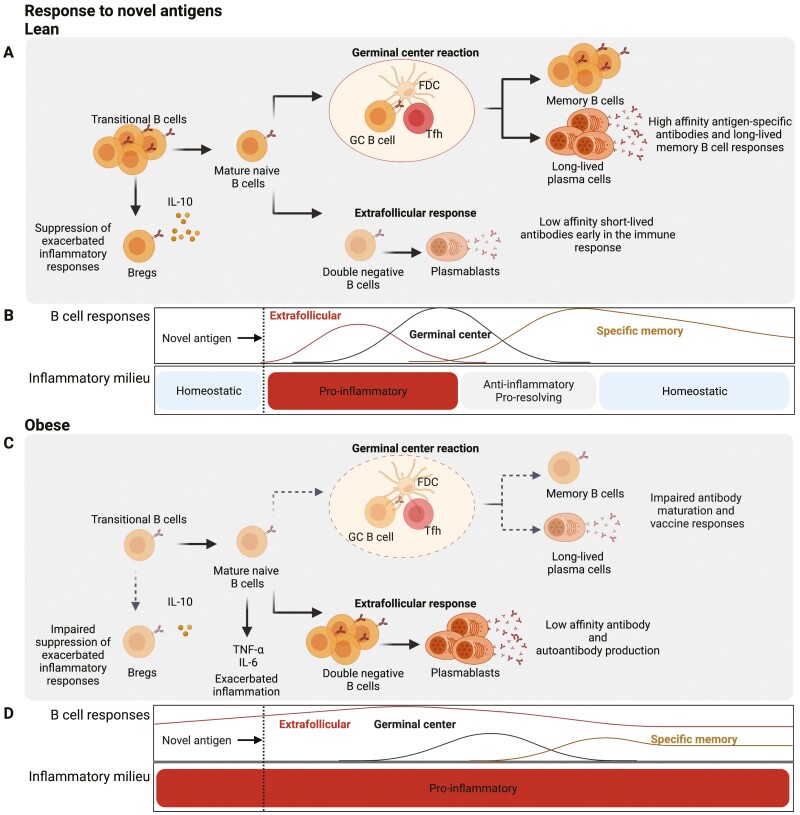
Proposed model for how obesity leads to impaired B cell responses to novel pathogens and vaccination. **(A)** In lean individuals, the response to pathogens is characterized by the early and short-lived extrafollicular response, associated with little somatic hypermutation, the process by which antibody affinity for encountered antigens increases. Subsequently, the germinal centre (GC) response gives rise to high affinity, long-lived antibody secreting cells (ASC) and memory B cells. The GC reaction is facilitated by antigen retention on follicular dendritic cells (FDC) and signals from T follicular helper (Tfh) cells. A subset of transitional B cells also acts to suppress exuberant inflammatory responses through the production of IL-10, which both restores homeostasis and limits chronicity. **(B)** Temporal cues from pro- and anti-inflammatory mediators guide the appropriate mounting of B cell responses to novel antigens in lean individuals—early extrafollicular (EF) response is followed by GC induction and finally culminates in the seeding and maintenance of the memory compartment. **(C)** In obesity, preferential B cell activation through the extrafollicular pathway leads to a depletion of available precursors for the GC reaction. This not only exacerbates autoantibody production by double negative (DN) B cells but also leads to a reduction of GC-derived plasma cells and memory B cells producing high-affinity neutralizing antibodies. The dysregulated extrafollicular response and severe inflammatory disease outcomes are further exacerbated due to a lack of IL-10-producing transitional B cells and an expansion of TNF-α- and IL-6-producing B cells. (**D**) The persistently high inflammatory profile in obesity inhibits the appropriate regulation of B cell responses to novel antigens, with continuous engagement of the EF pathway, minimal GC induction, and reduced memory. Created with BioRender.com.

Together these findings have renewed the interest of immunologists to understand how the new set-point established in the B cell compartment in people living with obesity alters the ability of these individuals to mount de novo responses. This will be a highlight of future research in obesity and is a critical consideration for potential future pandemics. As elucidated by human studies, individuals with obesity present with increased DN [36] and reduced transitional B cell frequencies in the absence of infection [40] among other defects (see Fig. 1). How do these pre-existing alterations in B cell responses underlie the increased severity of infection as well as impaired vaccine responses observed in these individuals (see Fig. 2)? This seeming paradox could be explained by the heightened chronic pro-inflammatory state that inhibits the appropriate mounting of adaptive immune responses. In other words, the chronicity of obesity-associated inflammation leads to dysregulated temporal cues that guide the induction and regulate the magnitude of adaptive immunity (see Fig. 2). For example, it has been shown that the induction of GC responses is preceded by a reduction in the extrafollicular (EF) response and that in chronic responses ongoing seeding of the EF pathway, decimates the GC response [85].

That B cell activation through the EF response, as observed in obese individuals in the absence of infection, is associated with more severe COVID-19 was demonstrated by two studies [86, 87]. Furthermore, patients that succumbed to SARS-CoV-2 infection lacked GCs and T follicular helper (Tfh) cells and exhibited a concurrent expansion of plasmablasts in lymph nodes and spleen [88]. Because DN B cells are expanded in obese individuals, it is tempting to speculate that this is exacerbated by infection leading to more severe COVID-19 due to an impaired generation of GC-derived plasma cells and memory B cells producing high-affinity neutralizing antibodies. Indeed, in SARS-CoV-2 infected individuals, a negative correlation between BMI and SARS-CoV-2-specific IgG has been reported [89]. An underlying reduction of Bregs may also contribute to increased severity in obese individuals. Indeed, severe COVID-19 is associated with the relative reduction in transitional B cells [86, 88], as also observed in individuals living with obesity [40].

What remains entirely unknown is whether alterations in adipose tissue B cells impact the ability of obese individuals to mount appropriate responses to SARS-CoV-2 infection. A recent study demonstrated that SARS-CoV-2 infects adipose tissue [90]. Zickler *et al*. studied SARS-CoV-2 dissemination to human adipose tissue in 30 individuals who died from COVID-19. In 10 of 18 male individuals, SARS-CoV-2 RNA was detected in adipose tissue; all of them were overweight or obese individuals [91]. Obesity-associated local subpar immunity could allow SARS-CoV-2 to spread to adipose tissue, serving as a viral reservoir. Interestingly, Reiterer *et al*. showed that SARS-CoV-2 infection induces adipose tissue dysfunction with reduced adiponectin and adiponectin-to-leptin ratio and this may lead to hyperglycaemia and insulin resistance [90], suggesting that inflammation can potentially act as a trigger of metabolic dysfunction.

## Future direction and concluding remarks

Obesity represents a global health crisis and the current COVID-19 pandemic poses a particular risk to people living with pre-existing conditions such as obesity. While previously the immune system and the adipose tissue were considered two independent biological systems, it is now clear that there is an extensive interplay between them. Importantly, the obesity-associated metabolic dysfunction and chronic low-grade inflammation appear to exacerbate metabolic abnormalities and alter immune responses. Both mechanistic investigations in murine models and evidence from human studies support the notion that B cells play an important role in this interplay. We support the model that in the lean state B cells act to maintain homeostasis of adipose tissue, creating an anti-inflammatory milieu, importantly through IL-10 and IgM. During the onset of obesity, the balance is shifted towards inflammatory B cell activation, which potentiates T cell responses and produces pathogenic antibodies contributing to both metabolic dysfunction and inflammation. Finally, the establishment of a new obesity-associated immune set-point with high inflammation precludes the regulation of response to novel antigens (see Fig. 2). Continued exploration of the B cell-related cellular and molecular mechanisms that contribute to morbidity and mortality in obese individuals is therefore crucial. Initial studies suggest a role for leptin, metabolic remodelling, changes to the gut-microbiota, and the availability of nutrients in the adipose tissue itself in driving potential changes to B cell phenotype. Among the important questions that remain to be addressed are: To what extent obesity-associated alterations in B cells can be reversed and corrected by weight loss? Is it possible to dissociate between weight and other metabolic factors in driving risk for infectious complications and reduced vaccination responses in people living with obesity? What is the adipose tissue B cell landscape in humans and how is it impacted by obesity? What are the exact mechanisms that drive B cell dysfunction in obesity? Answering these questions will enable better care and treatment options, including vaccination strategies, for the increasing number of people living with obesity.

## Data Availability

Not applicable.

## Abbreviations

AMPK: AMP-activated protein kinase
ASC: antibody-secreting cell;
BMI: body-mass index
Breg: regulatory B cell
DIO: diet-induced obesity
DN: double-negative
EF: extrafollicular
GC: germinal centre
HFD: high-fat diet
NCD: normal chow diet
ROS: reactive oxygen species
SAT: subcutaneous adipose tissue
Tfh: T follicular helper
VAT: visceral adipose tissue
WAT: white adipose tissue
